# Nucleotide Biosynthesis Is Critical for Growth of Bacteria in Human Blood

**DOI:** 10.1371/journal.ppat.0040037

**Published:** 2008-02-15

**Authors:** Shalaka Samant, Hyunwoo Lee, Mahmood Ghassemi, Juan Chen, James L Cook, Alexander S Mankin, Alexander A Neyfakh

**Affiliations:** 1 Center for Pharmaceutical Biotechnology, University of Illinois at Chicago, Chicago, Illinois, United States of America; 2 Section of Infectious Diseases, Immunology and Internal Medicine, Department of Medicine, University of Illinois at Chicago, Chicago, Illinois, United States of America; Yale University School of Medicine, United States of America

## Abstract

Proliferation of bacterial pathogens in blood represents one of the most dangerous stages of infection. Growth in blood serum depends on the ability of a pathogen to adjust metabolism to match the availability of nutrients. Although certain nutrients are scarce in blood and need to be *de novo* synthesized by proliferating bacteria, it is unclear which metabolic pathways are critical for bacterial growth in blood. In this study, we identified metabolic functions that are essential specifically for bacterial growth in the bloodstream. We used two principally different but complementing techniques to comprehensively identify genes that are required for the growth of Escherichia coli in human serum. A microarray-based and a dye-based mutant screening approach were independently used to screen a library of 3,985 single-gene deletion mutants in all non-essential genes of E. coli (Keio collection). A majority of the mutants identified consistently by both approaches carried a deletion of a gene involved in either the purine or pyrimidine nucleotide biosynthetic pathway and showed a 20- to 1,000-fold drop in viable cell counts as compared to wild-type E. coli after 24 h of growth in human serum. This suggests that the scarcity of nucleotide precursors, but not other nutrients, is the key limitation for bacterial growth in serum. Inactivation of nucleotide biosynthesis genes in another Gram-negative pathogen, Salmonella enterica, and in the Gram-positive pathogen Bacillus anthracis, prevented their growth in human serum. The growth of the mutants could be rescued by genetic complementation or by addition of appropriate nucleotide bases to human serum. Furthermore, the virulence of the *B. anthracis purE* mutant, defective in purine biosynthesis, was dramatically attenuated in a murine model of bacteremia. Our data indicate that *de novo* nucleotide biosynthesis represents the single most critical metabolic function for bacterial growth in blood and reveal the corresponding enzymes as putative antibiotic targets for the treatment of bloodstream infections.

## Author Summary

Bacterial growth in the bloodstream is a common manifestation of a number of bacterial infections. When growing in blood, bacteria not only have to evade the host's immune response, but also adjust their metabolism to suit availability of nutrients. Although the concentrations of various metabolites in human blood are known, it is difficult to predict which nutrients are abundant and which are scarce. To proliferate in human blood, bacteria need to synthesize metabolites that are present in the limiting concentrations. For that, they need to produce specific enzymes that are, thus, critical for the bacterial growth in the bloodstream. We carried out a comprehensive, genome-wide search for Escherichia coli genes that are essential for growth in human serum. We found that inactivation of nucleotide biosynthesis genes leads to a significant growth defect in human serum not only for E. coli but also for two other pathogens, *Salmonella* Typhimurium and Bacillus anthracis. The results of this study demonstrate that the limiting amounts of the nucleotide bases in human serum force invading pathogens to rely on *de novo* nucleotide biosynthesis. Hence, our findings reveal nucleotide biosynthesis enzymes as a possible target for the treatment of bloodstream infections.

## Introduction

Bacteremia, characterized by the presence of pathogenic bacteria in the bloodstream, is a major cause of morbidity and mortality worldwide. Bacteremia often leads to sepsis and death [1]. To survive in blood, bacterial pathogens must evade a multitude of host defense mechanisms such as complement-mediated lysis, phagocytosis and antimicrobial peptide-mediated killing. The spectrum of complement resistance mechanisms of bacteria is very wide and includes different activities like antigenic variation, use of membrane proteins to block binding of complement proteins and capsule biosynthesis [2–5]. Most extracellular pathogens avoid phagocytosis by synthesizing a protective capsule that helps to evade recognition [6,7]. Direct degradation of antimicrobial peptides and modification of cell surface properties are the major strategies used by bacteria to resist the bactericidal activity of host antimicrobial peptides like the platelet-derived thrombocidins in blood [8,9]. However, these immune-evasion strategies are mostly pathogen-specific and it is difficult to use the underlying mechanisms as targets for broad-spectrum antibiotics.

To proliferate in the various host niches that bacterial pathogens invade during the course of infection they need to adjust their metabolism to suit local nutrient availability. For example, the amount of free iron in human blood is 10^−18^ M [10]. Most pathogens need 10^−6^ to 10^−7^ M iron for growth and hence they use very complex and diverse strategies to acquire and store iron in order to grow in this iron-limiting environment of the host's blood [11]. The most common strategy of iron acquisition is the production of siderophores, high-affinity (10^30^ M^−1^) ferric iron-binding molecules that can sequester iron from the host's iron-binding proteins. Knockout of iron acquisition mechanisms has been shown to attenuate the virulence of many bacteria [12,13]. However, the uniqueness of blood as a niche for bacterial growth extends far beyond iron limitation: low availability of certain nutrients may define the ability of bacteria to proliferate in blood. Although the absolute abundance of various metabolites, such as amino acids and nucleotides, in human serum is known [14,15], it is unclear which nutrients are freely available and sufficient and which are limiting for bacterial growth in human serum.

Several previous reports described the importance of amino acid or nucleotide biosynthesis by bacteria in the cause of infection. For instance, certain auxotroph mutants of *Salmonella* [16–18], Staphylococcus aureus [19] or Streptococcus pneumoniae [20] have been shown to be avirulent in murine models of infection. These reports suggest that purines and some amino acids are scarce *in vivo*. Also, the inactivation of a potassium transporter in Vibrio vulnificus [21] or of a manganese, zinc and iron transporter in Streptococcus pyogenes [22] have been shown to attenuate virulence of the respective pathogens, suggesting that acquisition of some metal ions is critical for growth *in vivo*. Notwithstanding the fact that most of these genes were identified in non-exhaustive screens, these studies only provide evidence of the limited availability of the corresponding metabolites in the host. They do not describe nutrient availability in various host compartments invaded by the pathogens during infection. It is unclear whether the reduced virulence of these mutants can be attributed to their inability to grow specifically in blood. The comprehensive identification of genes that are critical, specifically for the bloodstream growth of pathogens, has never been attempted. Hence, crucial nutrient requirements that need to be fulfilled during bacterial growth in blood are essentially unknown.

Identification of the limiting nutrients and of bacterial genes that are critical for growth in blood can pinpoint biosynthesis and acquisition strategies that are crucial during the bacteremic stage of infection. Enzymes critical for survival and proliferation of pathogenic bacteria in blood can provide potential targets for treatment of bloodstream infections.

To this end, we sought to identify genes required for the growth of bacteria in human blood. We screened a comprehensive gene-deletion library of the model Gram-negative organism, Escherichia coli, for mutants unable to grow in human serum. We found that *de novo* purine and pyrimidine biosynthesis is the key pathway critical for E. coli growth in serum, thereby revealing the limited availability of nucleotide precursors as the major limitation for bacterial growth in blood. Salmonella enterica, an important Gram-negative pathogen, exhibited a similar requirement for *de novo* biosynthesis of purines and pyrimidines for growth in serum. Deletion of the corresponding genes in the evolutionarily distant Gram-positive pathogen Bacillus anthracis demonstrated the universal need for these two biosynthetic pathways for bacterial growth in serum.

## Results

### Biosynthesis of Purines and Pyrimidines Is Critical for the Growth of E. coli in Human Serum

Our major goal was to identify genes that are critical for the survival and growth of bacteria in blood. Specifically, we were interested in identifying genes that mediate adaptation to the unique nutrient composition of blood rather than those which facilitate immune evasion. Hence, in our experiments we used human serum in which the function of the complement system was inactivated by heat-treatment.

E. coli is a major cause of Gram-negative bacteremia in hospitalized patients [23]. We used E. coli as an experimental model for our initial experiments. Specifically, we used a defined library of 3985 E. coli single-gene deletion mutants (“Keio collection”), where all non-essential genes of an E. coli K12 laboratory strain BW25113 have been individually replaced by a kanamycin-resistance cassette [24].

For the identification of genes required for the growth of bacteria in serum, we employed a genetic technique called MGK (Monitoring of Gene Knockouts) [25]. MGK is a microarray-based approach that uses the chromosomal flanks of inactivated genes as hybridization targets for custom-designed oligonucleotide microarrays. It allows simultaneous assessment of the relative abundance of thousands of mutants in a population and identification of genes whose inactivation is unfavorable for cell growth under selective conditions.

**Figure 1:**
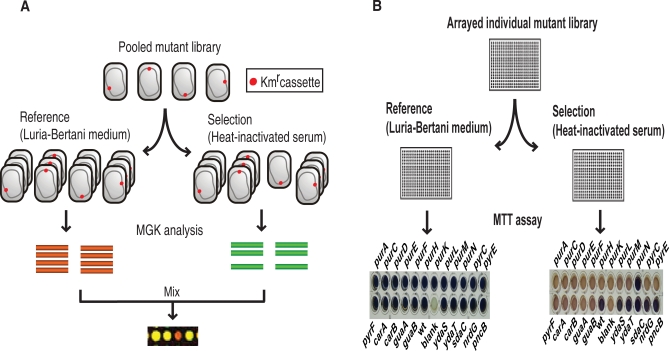
Identification of Genes Required for the Growth of E. coli in Human Serum (A) Schematic representation of the identification of E. coli mutants from the Keio library with a growth defect in human serum using Monitoring of Gene Knockouts (MGK). (B) MTT dye-based screening for mutants with a growth defect in human serum. MTT was added after 24 h of growing the arrayed mutant library in 96-well plates in either LB medium or serum. Mutants that could grow in serum generated a deep blue color by reducing MTT. The lack of a blue color in a serum bacterial culture served as a qualitative indicator of the inability of that mutant to grow in serum.

To apply MGK, mutants in the Keio collection were individually grown and mixed at a similar ratio (see Protocol S1 for details). The pooled library was grown in either serum or in LB for 5 h (approximately 4 generations in serum) (Figure 1A). Mutants lacking genes critical for growth in blood are expected to be underrepresented in the resulting population of cells grown in serum. Harvested cells were allowed to re-grow in fresh LB medium in order to enrich the population for viable cells and minimize isolation of genomic DNA from dead cells. “MGK targets” corresponding to the flanks of inactivated genes were generated as described in Protocol S1 using genomic DNA isolated from cells grown in the reference (LB) and the selective (serum) conditions (Figure 1A), and co-hybridized to an oligonucleotide microarray. Two independent MGK experiments (with dye-swapping) were performed.

**Figure 2:**
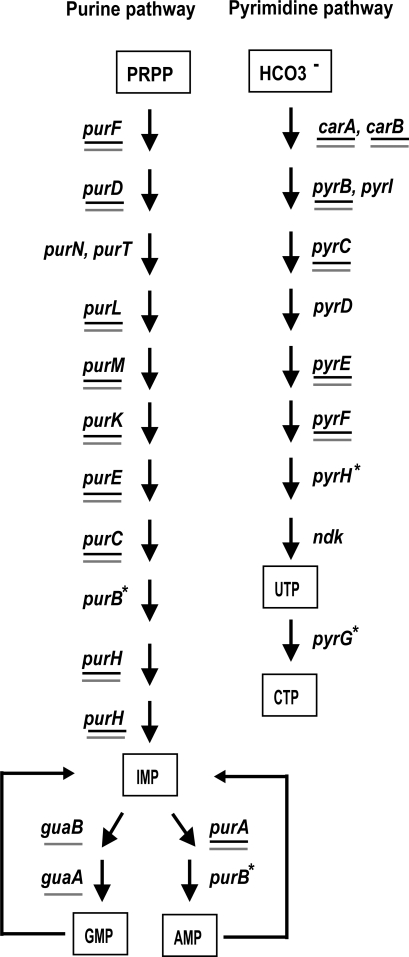
Genes Required for the Growth of E. coli in Human Serum Identified by MGK and MTT Assay Purine and pyrimidine nucleotide biosynthesis pathways in E. coli are outlined. Genes that were identified to be essential for the growth of E. coli in human serum by the MGK approach are underlined in black and those identified by the MTT assay are underlined in grey. Essential genes, whose deletions are absent from the defined library of E. coli mutants, are marked by an asterisk. Genes whose deletion does not confer a growth defect in serum are *pyrI*, *ndk*, *purN* and *purT*. The *pyrD* mutant was not identified by either screen, although it showed a growth defect in human serum.

Twenty-two mutants with a potential growth defect in serum were identified that consistently showed at least a 2-fold reduction in the hybridization signal of the serum sample as compared to the LB sample. Strikingly, the majority of these mutants (15 out of 22) carried a deletion of a gene involved in biosynthesis of either purines or pyrimidines (Table 1 and Figure 2). This result suggested that the *de novo* biosynthesis of purines and pyrimidines is crucial for the growth of E. coli in human serum and that the scarcity of nucleotide precursors is the major limiting factor for bacterial growth in blood.

**Table 1:**
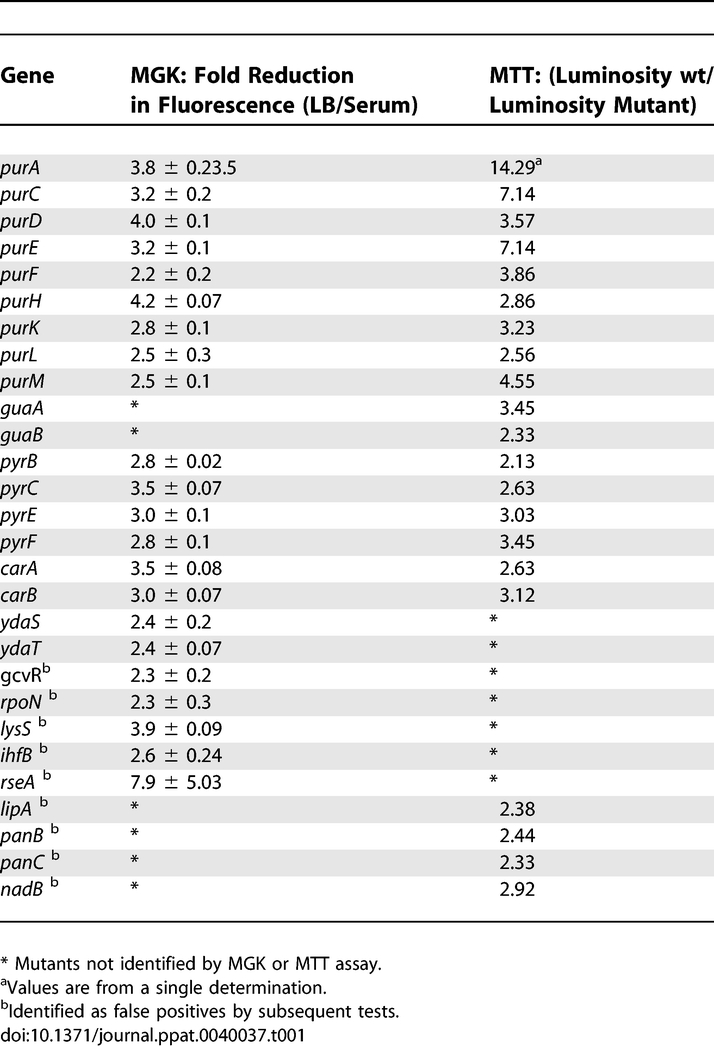
Mutants with a Growth Defect in Serum Identified by MGK and MTT Dye-Based Colorimetric Screening of the Keio Collection of E. coli Mutants

In an MGK experiment, thousands of mutants are grown together in a mixed population and growth characteristics of each mutant can be potentially affected by metabolites secreted by other cells. In addition, during re-growth in LB media, the mutants that had growth defects in serum could potentially catch up with the rest of the cells. To exclude this scenario, we supplemented the MGK approach with an independent screen involving replica growing of the 3,985 individual mutants from the Keio collection, arrayed in a 96-well format, in serum and LB. The inherent turbidity of serum prevents the use of optical density as a measure of bacterial growth. Therefore, we used a dye, 3-(4,5-dimethylthiazol-2-yl)-2,5-diphenyltetrazolium bromide (MTT), to detect viable cells [26,27]. After overnight incubation of mutants in 96-well plates in LB or in human serum, MTT dye was added to the wells and the plates were incubated for another 4–5 h at 37°C. Overnight incubation of the cells in human serum before addition of the MTT dye is expected to identify mutants that are severely impaired in growth in serum rather than mutants that show only modest growth defects. Live, actively growing bacteria reduce MTT to a blue formazan precipitate resulting in a deep blue color of the live serum cultures. The lack of a blue color served as a qualitative indicator of the inability of that mutant to grow in serum (Figure 1B).

The MTT screen identified 21 mutants that failed to grow selectively in serum (Table 1 and Figure S1A). Of these, 17 mutants (81%) carried deletions of genes involved in the nucleotide biosynthesis pathway. Fifteen of these 17 mutants were also identified by the MGK analysis (Figure 2).

**Figure 3:**
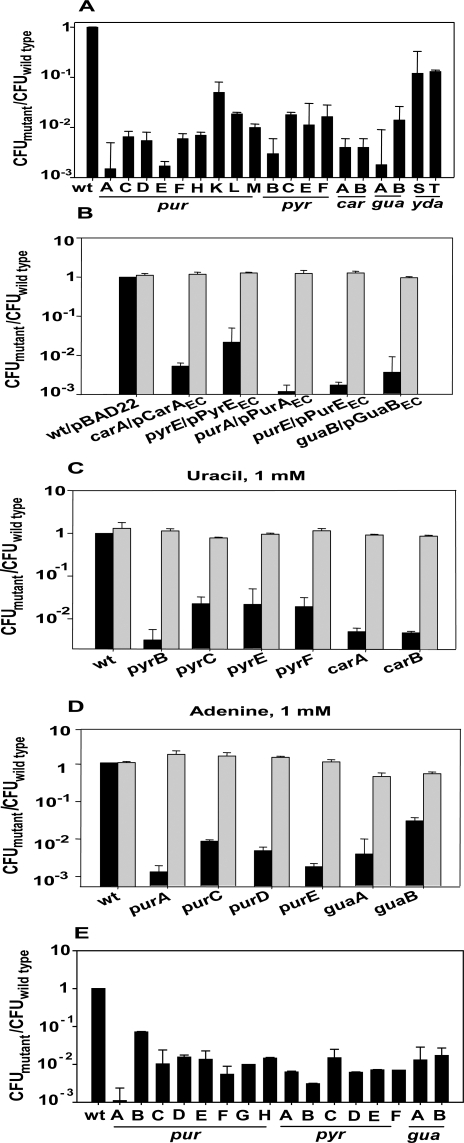
E. coli and *S. typhimurium pur* and *pyr* Mutants Have a Growth Defect in Human Serum (A) Ratios of the viable cell counts of 19 mutants identified by either MGK or MTT assay as compared to wild-type E. coli after 24 h of growth in human serum. *p* Values for each mutant relative to wild type were lower than 0.001. (B) Ratios of the viable cell counts of *carA*, *pyrE*, *purA*, *purE* and *guaB* mutants (black bars) and the viable cell counts of *carA*/pCarA_EC_, *pyrE*/pPyrE_EC_, *purA*/pPurA_EC_, *purE*/pPurE_EC_, *guaB*/pGuaB_EC_ and wt+pBAD22 (grey bars) to those of wild-type E. coli following 24 h of growth in serum. (C) Grey bars represent ratios of the viable cell counts of each pyrimidine mutant to that of the wild-type E. coli after 24 h of growth in serum supplemented with 1 mM uracil. Black bars represent these ratios in serum without any nucleotide supplements. (D) Grey bars represent ratios of the viable cell counts of each purine mutant to that of the wild-type E. coli after 24 h of growth in serum supplemented with 1 mM adenine. Black bars represent these ratios in serum without any nucleotide supplements. (E) Ratios of the viable cell counts of Tn5 transposon insertion mutants in several *pur* and *pyr* genes of S. typhimurium to that of the wild type determined after 24 h of growth in human serum by plating serial dilutions on LB agar plates are represented by vertical bars. *p* Values for each mutant relative to wild type were lower than 0.01. Error bars indicate standard deviation (SD) for two independent experiments. Statistical significance of the data was calculated using *t*-test in EXCEL.

To verify the phenotypes of E. coli mutants identified by the MGK and MTT screens, we followed their survival and growth in human serum by determining the number of colony forming units (cfu). This rigorous verification showed that mutants which lacked genes belonging to pathways other than purine or pyrimidine biosynthesis (*gcvR*, *rpoN*, *lysS*, *ihfB* and *rseA* identified only by the MGK analysis and *nadB*, *panB*, *panC*, *iscS* identified only by the MTT assay) were apparently false positives. Two mutants, *ydaS* and *ydaT*, carrying deletions in genes with unknown function, found only in the MGK screen, exhibited a mild growth defect (∼10-fold reduction in cfu) and were not studied further. Notably, however, all mutants carrying a deletion in one of the *pur* or *pyr* genes showed a significant (20- to ∼1,000-fold) reduction in the viable cell number compared to wild-type E. coli after 24 h growth in serum (Figure 3A). These included 15 *pur* or *pyr* mutants identified by both MGK and MTT screens and two *pur* mutants (*guaA* and *guaB*) identified only by the MTT screen (Figure 2). Importantly, after incubation in serum, the cfu counts of some mutants dropped below the initial inoculum suggesting that these mutants not only had a growth defect, but were actively dying in serum (Figure S1B). All *pur* and *pyr* mutants grew well in LB medium, indicating that the growth defect was serum-specific (Figure S1B). These results suggest that nucleotide biosynthesis is a critical metabolic function required for growth of E. coli in human serum and that the scarcity of nucleic acid precursors, but not other metabolites, is the key metabolic limitation for bacterial growth in human blood.

Some of the E. coli nucleotide biosynthesis genes are organized in operons. Therefore, their replacement by the gene inactivation cassette could have a polar effect on the expression of downstream genes. We tested and eliminated this possibility by genetic-complementation studies. Structural regions of three operon-encoded E. coli genes *carA*, *pyrE*, and *guaB* (the first genes in the respective operons) (Figure S1C), along with ∼300 bp upstream regions that include their native promoters, were cloned on a suitable vector and introduced into corresponding mutant strains. The genetically complemented E. coli strains (*carA*/pCarA_EC_, *purE*/pPurE_EC,_ *guaB*/pGuaB_EC_) thus obtained (Table S1) were tested for growth in serum. Similarly complemented two strains of gene deletions corresponding to monocistronic operons, *purA* and *pyrE* (*purA*/pPurA_EC_ and *pyrE*/pPyrE_EC_) were also tested. All complemented E. coli mutant strains grew as well as the wild type in serum (Figure 3B). This confirmed that the observed growth defect of the E. coli mutants in serum was due to the lack of the corresponding gene and not the manifestation of polar effects of gene replacement on downstream genes.

In order to determine whether the observed growth defect of the E. coli mutants in serum was indeed due to a limiting supply of nucleic acid precursors, mutant growth was tested in serum supplemented with an appropriate nucleobase, either adenine or uracil, for the purine or pyrimidine biosynthesis mutants respectively. Addition of the appropriate nucleobase to serum rescued the growth of the mutants (Figure 3C and 3D). This result confirmed that the purine and pyrimidine deficiency of human blood forces invading bacteria to rely on the *de novo* biosynthesis of these metabolites.

### Nucleotide Biosynthesis Pathways Are Critical for the Growth of Salmonella enterica Serovar Typhimurium in Human Serum

Virulent E. coli strains are extracellular enteric pathogens. In contrast, the Gram-negative pathogen, Salmonella enterica serovar Typhimurium (S. typhimurium), can also replicate intracellularly in phagocytes. Previous studies have shown that inactivation of nucleotide biosynthesis genes in S. typhimurium attenuates its virulence [16,28,29]. This effect has been attributed solely to the inability of the mutants to colonize the intracellular niche. Our finding that the inactivation of nucleotide biosynthesis genes prevents E. coli from growing in human serum prompted us to test whether *Salmonella* strains defective in purine or pyrimidine biosynthesis show growth defects in serum. We tested the growth in human serum of 14 different S. typhimurium mutants in which *pur* or *pyr* genes were inactivated by transposon insertions (Table S1). Most of the mutants showed a substantial growth defect with 10- to ∼100-fold or more reduction in viable cell counts after 24 h of growth in human serum as compared to the wild-type strain (Figure 3E). This result demonstrated that *de novo* nucleotide biosynthesis is required for growth of S. typhimurium in human serum and that the attenuated virulence of such mutants may be due, at least in part, to an inability of the pathogen to multiply in the host's blood.

### Gram-Positive *Bacillus anthracis* Requires Nucleotide Biosynthesis for Growth in Human Serum

The systemic stage of the life-threatening anthrax infection is characterized by the rapid growth of B. anthracis in the host's blood reaching up to10^8^ bacteria/ml [30]. Few treatment options are available for the late stages of anthrax infections. Thus, it was of particular interest to investigate whether purine and pyrimidine biosynthesis is critical for the growth of Gram-positive B. anthracis in human serum, as was observed for Gram-negative E. coli and S. enterica.

**Figure 4:**
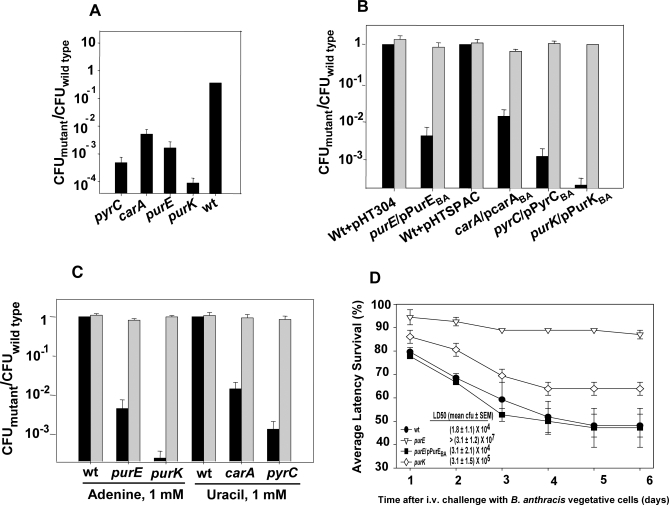
B. anthracis Nucleotide Biosynthetic Mutants Are Unable to Grow in Human Serum (A) Ratios of the viable cell counts of the *carA*, *pyrC*, *purE* and *purK* mutants as compared to wild-type B. anthracis after 24 h of growth in human serum (*p* value <1 × 10^−5^). Statistical significance of respective mutants relative to wild type was calculated using *t*-test in EXCEL. (B) Ratios of the viable cell counts of *purE*, *carA*, *pyrC* and *purK* mutants (black bars) and the viable cell counts of *purE*/pPurE_BA_, *carA*/pCarA_BA_, *pyrC*/pPyrC_BA_, *purK*/pPurK_BA_, wt + pHT304 and wt + pHTSPAC (grey bars) to that of wild-type B. anthracis following 24 h of growth in serum. (C) Grey bars represent ratios of the viable cell counts of each mutant to that of wild-type B. anthracis after 24 h of growth in serum supplemented with 1 mM adenine (purine mutants) and 1 mM uracil (pyrimidine mutants). Black bars represent these ratios in serum without any nucleotide supplements. Error bars indicate SD of triplicate experiments. (D) Attenuation of *B. anthracis purE* mutant in a murine model of bacteremia. Survival curves (Average Latency Survival) were derived from transformation of daily LD_50_s estimated for mouse cohorts challenged intravenously with a dose range (10^3^–10^8^) of vegetative cells of the indicated B. anthracis strains. Points on survival curves represent the mean ± SEM values from three (Sterne, *purE*) or two (*purE*/pPurE_BA_, *purK*) different mouse experiments. LD_50_ values shown in the graph represent the final values estimated at the end of the 6-day observation periods. Survival results were unchanged when mice were observed for an additional 2 weeks.

Mutants with deletions in either *pur* (*purE* and *purK*) or *pyr* (*carA* and *pyrC*) genes were constructed in B. anthracis Sterne 34F2 strain (pXO1^+^ pXO2^−^) by allelic gene replacement. In agreement with the observations made with Gram-negative pathogens, all of the B. anthracis mutants displayed a severe growth defect in human serum: a 50- to ∼1,000-fold decrease in viable cell counts as compared to the wild type after 24 h (Figure 4A). None of these mutants showed reduced growth in LB medium (data not shown). Introduction of the plasmid carrying the deleted gene or addition of appropriate nucleobases rescued growth of these mutants in serum (Figure 4B and 4C and Table S1).

This result demonstrates that the limiting amounts of purines and pyrimidines in serum restrict the growth of B. anthracis mutants and shows that *de novo* nucleotide biosynthesis is a conserved requirement for the growth of at least three bacterial species in human serum.

### Inactivation of *purE* Significantly Attenuates Virulence of *B. anthracis*

We hypothesized that the growth defect exhibited by B. anthracis nucleotide biosynthesis mutants in serum would manifest at the bacteremic stage of the infection resulting in attenuated virulence. We used a murine model of anthrax infection to test this hypothesis. For these experiments we employed the B. anthracis Sterne strain, which causes lethal infections in certain strains of inbred mice with pathologies similar to systemic anthrax infection in humans [31,32].

Murine serum contains about 30-fold more cytidine as compared to human serum (3 μM compared to 0.1 μM, respectively) [14]. Therefore, it was not surprising that unlike in human serum, the pyrimidine biosynthesis mutants of B. anthracis did not show any strong growth defect in murine serum. However, as expected, purine biosynthesis mutants *purE* and *purK* were defective for growth in murine serum (Figure S3). The virulence of these two mutants was tested in a murine infection model in which mice were challenged by direct intravenous inoculation with increasing numbers of bacilli and observed for survival following this experimental bacteremia. The *purE* knockout mutant showed a dramatic decrease in virulence as evidenced by ∼3.5 log increase in LD_50_ (*p* = 0.002) and an increased survival of the challenged mouse cohort (*p* < 0.001) as compared to mice challenged with the wild-type Sterne strain (Figure 4D). Mice challenged with the *purE* mutant remained healthy for the entire additional 2-week period of observation and no bacteria could be cultured from their blood or spleen. In contrast, a *purE* mutant strain complemented with a plasmid carrying the *purE* gene (*purE*/pPurE_BA_), was as virulent as the wild-type Sterne strain. Unexpectedly, the *purK* mutant, which exhibited a similar growth defect in serum *in vitro* as the *purE* mutant was almost as virulent as the wild-type Sterne strain (Figure 4D). This result showed that *purE*, but apparently not *purK*, is essential for virulence of B. anthracis in mice and thus reveals PurE as a putative antibiotic target for treatment of anthrax bacteremia.

## Discussion

In this paper we demonstrate that *de novo* nucleotide biosynthesis is critical for survival and growth of bacteria in human serum. A near exhaustive search using two independent screening strategies based on co-growth of E. coli gene knockout mutants (MGK) and analysis of individual mutants (MTT assay) applied to a comprehensive library of E. coli mutants consistently pointed to the importance of mainly *pur* and *pyr* genes for E. coli growth in human serum. The overlap of the results of both screens identified 15 *pur* or *pyr* genes that are required for successful growth of E. coli in human serum (Figure 2). This result was confirmed for two other pathogenic species of bacteria, Gram-negative S. typhimurium and Gram-positive B. anthracis.

Inactivation of most of the *pur* and *pyr* genes was detrimental to bacterial growth in serum. Of the 13 non-essential genes involved in the purine biosynthetic pathway in E. coli, only two genes, *purN* and *purT*, were not identified as being critical for growth in human serum (Figure 2). This is not surprising because *purN* and *purT* both encode 5'-phosphoribosylglycinamide transformylases with partly overlapping specificities [33], and their individual inactivation should have little effect on cell growth. Of the 9 non-essential genes involved in the pyrimidine biosynthetic pathway our screens did not identify *pyrI*, *pyrD,* and *ndk* mutants. Upon checking the growth of the *pyrD* mutant in serum, we observed a significant growth defect similar to that of the mutants identified by the two screens (Figure S2A). The other two mutants did not show strong phenotype in serum (Figure S2A). This result was expected because *pyrI* encodes the regulatory subunit of the aspartate carbamoyltransferase that is not critical for the function of the enzyme [34], while Ndk is a nucleoside-diphosphate kinase whose function is partly redundant [35].

Iron acquisition genes are known to be important for bacterial adaptation to growth in the iron-limiting environment of blood [10]. Yet no such mutants were identified in our screens, and testing of several such individual E. coli mutants (*entA*, *fepE*, *fecA*, and *tonB*) showed that their growth in serum was not impaired (Figure S2B). One possible explanation for this result is that heat inactivation destroys transferrin-iron complexes and releases free iron that can be used by bacteria.

The virulence of *B. anthracis pur* mutants has been previously characterized in a murine peritoneal cavity infection model [36]. Of all the mutants tested by Ivanovics *et al*, only those lacking PurA or PurB activity were found to be significantly attenuated in mice. On the other hand, our study shows that the *B. anthracis purE* mutant is significantly attenuated in virulence in a murine bacteremia model. These results suggest that only certain enzymes in the purine biosynthetic pathway can be potential targets for antibiotics. The observed attenuation of the *B. anthracis purE* mutant not only reveals the PurE enzyme as a novel target for the development of anti-anthrax therapies, but also indicates that deletion of *purE* in a fully virulent B. anthracis strain could be a promising way to develop a live attenuated vaccine. In contrast to the *purE* knockout strain, the *purK* mutant of B. anthracis remained virulent and killed mice with an LD_50_ that was only slightly higher than that of the wild-type Sterne strain (Figure 4D). This result is not too surprising. PurK catalyses the carboxylation of aminoimidazole ribonucleotide (AIR) leading to an unstable intermediate, N^5^-carboxyaminoimdazole ribonucleotide (N^5^-CAIR) [37,38], which is then converted by PurE to carboxyaminoimidazole ribonucleotide (CAIR). In vitro studies showed that a significant fraction of AIR can be non-enzymatically converted to N^5^-CAIR at a high concentration of bicarbonate [39]. The small amounts of N^5^-CAIR produced spontaneously in the *purK* mutant might relieve the block in purine biosynthesis and rescue the auxotrophy [40].

Our data strongly suggest that nucleotide biosynthesis is the key metabolic pathway which is critical for proliferation of bacterial pathogens in blood. It might be argued that our conclusions could be deduced empirically from a known low concentration of free nucleobases in the human blood serum. Indeed, some previous studies pointed to a relative scarcity of purines in blood [19]. However, other studies suggested that purines and pyrimidines are sufficiently abundant in this niche [41] and the importance of nucleotide prototrophy for bacterial growth in human blood has never been clearly demonstrated. Furthermore, the simple knowledge of the nutrient composition of blood serum is not sufficient to predict which biochemical pathway will be rate-limiting for bacterial growth in this niche. Thus, although threonine, lysine, histidine, aromatic amino acid and riboflavin biosynthetic functions have been shown to be important for bacterial infections, none of these pathways appears to be critical for growth of bacteria in blood [19,42,43].

Several previous studies have shown that pathogens require nucleotide biosynthesis to establish a successful infection [16,19,20,44,45]. Our study, however, is the first to our knowledge to demonstrate the importance of the *de novo* synthesis of purines and pyrimidines for successful proliferation of pathogens specifically in blood. Reproduction of the phenotypes associated with several identified gene knockouts in Gram-negative bacteria, E. coli and S. typhimurium, in a Gram-positive pathogen B. anthracis, suggests the universal importance of the nucleotide biosynthesis pathways for growth of bacteria in the bloodstream. Indeed, as our data shows, nucleotide biosynthesis may be the only metabolic pathway that is universally required by bacterial pathogens invading the blood. The corresponding enzymes thus appear as putative antibiotic targets for curbing bacterial growth in the bacteremia stage of infection. Of the enzymes identified in this study as essential for growth of bacterial pathogens in human blood, two, PyrC and PurE, are especially attractive as targets for antibiotics. PyrC is a dihydroorotase that catalyzes the reversible cyclization of carbamoyl aspartate to dihydroorotate [46]. The known dihydroorotases fall into two major sequence classes. Class I enzymes are conserved among fungi and most Gram-negative proteobacteria whereas higher eukaryotes use class II dihydroorotases [47]. The limited sequence conservation between the two types of PyrC (less than 20%) [48] makes this enzyme an attractive target for the treatment of Gram-negative and fungal infections. PurE shows a higher degree of overall conservation between bacteria and eukaryotes (Table S2). However, the catalytic mechanisms of the bacterial and eukaryotic PurE enzymes are substantially different. In bacteria, PurE utilizes N^5^-CAIR to make CAIR, whereas human PurE uses AIR and CO_2_ and does not recognize N^5^-CAIR as a substrate [49], pointing to a significant difference in the structure of the catalytic centers in the bacterial and human enzymes. Given that the *B. anthracis purE* mutant is avirulent, apparently due to inability of bacteria to proliferate in blood, PurE emerges as an attractive target for antibiotic therapies.

Nucleotides are important substrates not only for DNA synthesis but also for DNA repair. Thus inhibitors targeting the nucleotide biosynthesis functions identified in this study can also impede the repair processes following bacterial DNA damage induced by the host's reactive oxygen intermediates during infection.

Detailed exploration of PyrC and PurE as well as other enzymes of the nucleotide biosynthesis pathways as potential antibiotic targets may lead to development of new therapies for treatment of bacterial bloodstream infections.

## Materials and Methods

### Bacterial strains, plasmids, and growth conditions.

The collection of gene knockout mutants of the E. coli strain BW25113 (Keio collection) was obtained from Nara institute of Science and Technology, Japan [24]. Wild-type Salmonella enterica serovar Typhimurium LT2 and isogenic Tn5 transposon insertion mutants were obtained from the Salmonella Genetics Stock Center (Alberta, Canada) (http://www.ucalgary.ca/~kesander/). B. anthracis Sterne (pXO1^+^, pXO2^−^) wild-type strain [50] was used to construct *pur* and *pyr* biosynthesis mutants. All the cloning procedures were carried out using the OneShot TOP10 chemically competent E. coli cells (Invitrogen) as the host. E. coli strain GM 2163 (New England Biolabs) was used to obtain unmethylated plasmid DNA for transformation of B. anthracis.

The vector pKS1 was used to construct deletion mutants in B. anthracis [51]. Genetic complementation was carried out using recombinant plasmids based on the pBAD22 vector for E. coli or pHT304 vector [52] and pHTPSAC vector (H. Lee, unpublished data) for B. anthracis. Construction of the plasmids used for genetic complementation is outlined in Protocol S1. All primers used in this study are listed in Table S3.

For all experiments involving growth of bacteria in serum, frozen serum [Sterile filtered type-AB human serum, Cat No. H4522 (Sigma)] was thawed at 37°C, heat-inactivated by incubation for 30 min at 56°C and buffered at pH 7.0 by addition of 1 M HEPES buffer (pH 5.2) to the final concentration of 5 mM. Control cultures were grown in Luria-Bertani medium [53]. When necessary, antibiotics were added at the following final concentrations: kanamycin 30 μg/ml for E. coli or 100 μg/ml for B. anthracis, erythromycin 200 μg/ml for E. coli or 5 μg/ml for B. anthracis and ampicillin 100 μg/ml for E. coli.

### MGK selection and analysis.

The MGK selection was carried out essentially as described, by Smith *et al* [25], with minor modifications specified in Protocol S1.

### MTT dye-based testing.

Mutants from the Keio collection were replicated into 96-well plates containing 100 μl/well of LB supplemented with kanamycin. Plates were incubated overnight at 37°C with shaking. Cells were pelleted, washed once with PBS and resuspended in 100 μl PBS. 1 μl culture from each well was inoculated into fresh 96-well plates containing either LB or heat-inactivated human serum and incubated overnight with shaking at 37°C. The next day, 10 μl of 0.5% dye solution [MTT; (3-(4,5-dimethylthiazol-2-yl)-2,5-diphenyltetrazolium bromide) in PBS] was added to each well and the plates were incubated for 4–5 h at 37°C. When quantitation was required, plates were scanned using a Hewlett Packard Scanjet 5300C and Adobe Photoshop was used to determine luminosity values in each well (Figure S1A).

### Testing of the B. anthracis mutants in the murine infection model.

B. anthracis Sterne strain, the *purE* and *purK* knockout mutants, or genetically complemented mutant *purE*/pPurE_BA_ (Table S1) were grown in LB media, washed three times with PBS, and resuspended in PBS at a cell density of ∼5x10^8^ cfu/ml. Cohorts of adult (8–10 weeks old), female, NIH-Swiss mice, obtained from Frederick Cancer Research Center, were inoculated via tail vein with 0.2 ml of PBS containing serial 10-fold dilutions of vegetative B. anthracis cells (10^3^-10^8^ cfu/mouse; 4–6 mice per bacterial dose in the studies represented). Mice were observed daily for 6 days for signs of fatal outcome, using humane end points approved by the UIC institutional animal care and use committee. To confirm that the fatal infection was caused by an inoculated strain, the presence of the correct gene knockout was verified by PCR analysis of the bacterial colonies recovered from blood or spleen of diseased animals. 50% lethal dose (LD_50_) was estimated using the Spearman-Karber method [54]. The LD_50_ data were converted to average latency survival (ALS) curves using the described transformation [55]. Data were analyzed using SigmaPlot software and log rank test of the significance of differences in survival curves and *t*-test analysis of the significance of differences in LD_50_s among animal cohorts challenged with different bacterial strains.

## Supporting Information

Figure S1 Identification and Characterization of E. coli Mutants with a Growth Defect in Human Serum (A) A Hewlett Packard 5600C Scanjet was used to scan the 96-well plate in Figure 1B after addition of 10 μl of MTT dye following 24 h of growth in human serum. Luminosity values corresponding to each well were determined using Adobe Photoshop. Ratios of corrected luminosity (individual luminosity − background luminosity) for each mutant to corrected luminosity for wild type were determined for three independent experiments. *p* Values for all *pur* and *pyr* mutants were lower than 0.001. Statistical significance of respective mutants relative to wild type was calculated using *t*-test in EXCEL. Background luminosity is determined from a well with only medium to which MTT dye was added. Error bars indicate standard deviation of triplicate experiments.
(B) Growth curves for four E. coli mutants, *purK* (open circles), *purL* (filled inverted triangles), *carA* (open triangles) and *carB* (filled squares) as compared to wild type (filled circles) in LB medium and human serum. Viable counts were determined at regular time points by plating serial dilutions of each culture.
(C) Organization of the *carA* and *pyrE* (pyrimidine biosynthesis) and *purA*, *guaB* and *purE* (purine biosynthesis) genes on the E. coli genome.
(1.4 MB TIF)

Figure S2 Phenotype of Some E. coli Mutants Not Identified either by MGK or MTT Assays Ratios of the viable cell counts of the (A) *pyrD*, *pyrI* and *ndk* deletion mutants of E. coli BW25113 and (B) the *tonB*, *entA*, *fecA* and *fepE* deletion mutants of E. coli BW25113 to that of the wild-type parent are shown after 24 h of growth in human serum.
(328 KB TIF)

Figure S3 *B. anthracis purE* and *purK* Mutants Are Unable to Grow in Murine Serum (A) *carA*, *pyrC*, *purE* and *purK* mutants and wild-type B. anthracis grown in murine serum or human serum for 24 h in 96-well plates and stained with MTT. Control wells contain murine or human serum with no cells.
(B) Ratios of the viable cell counts of the *carA*, *pyrC*, *purE* and *purK* mutants of B. anthracis to that of the wild-type cells after 24 h growth in murine serum.
(2.0 MB TIF)

Protocol S1 Supporting Materials and Methods Identification of E. coli mutants defective for growth in human serum by MGK; construction of gene knockout mutants in B. anthracis; verification of phenotypes of individual mutant strains; and construction of plasmids for genetic complementation.
(59 KB DOC)

Table S1 Plasmids and Strains Used in This Study (88 KB DOC)

Table S2 Amino Acid Sequence Similarity of the B. anthracis and Homo sapiens Enzymes to the E. coli Nucleotide Biosynthesis Enzymes Required for Growth in Blood Serum (48 KB DOC)

Table S3 Primers Used in This Study (60 KB DOC)

## Abbreviations

*pur*: purine biosynthesis
*pyr*: pyrimidine biosynthesis
