# Supplementary material for: Nucleotide Biosynthesis Is Critical for Growth of Bacteria in Human Blood
Source: PLoS Pathog. 2008 Feb 15;4(2):e37. doi: 10.1371/journal.ppat.0040037 (PMC2242838; doi:10.1371/journal.ppat.0040037)
Supplement: Supplementary file 1 [file ppat.0040037.sd001.doc]

**MATERIALS AND METHODS**

**Identification of *E. coli* mutants defective for growth in human serum by MGK**

*Growth of mixed library*

All incubations were performed aerobically at 37^o^C with shaking. The Keio collection consists of 3985 single-gene deletion mutants constructed in the parent *E. coli* K-12 strain BW25113 arrayed in a 96-well format. In each deletion strain, the coding region of a non-essential gene (except for 7 C-terminal codons) has been replaced with a kanamycin resistance marker [1]. For the MGK selection, all 3985 mutants were grown individually in 96-well deep plates overnight at 37^o^C to an OD_600_ ~1.3 in LB with 30 μg/ml kanamycin. Equal volumes of each culture were combined together, cells were harvested from this combined culture, washed with LB and resuspended in LB supplemented with 15% glycerol. 500 μl aliquots containing ~ 10^9^ cells/ml were frozen and stored at -80^o^C (Keio library mix). An aliquot of the Keio library mix was diluted into 100 ml LB medium to OD_600_ 0.04 and grown to OD_600_ 0.4. Cells were harvested, washed once with PBS and diluted to OD_600_ ~ 0.04 into 100 ml of serum or LB. After 5 h incubation, cells from each growth condition were harvested, washed once with PBS, resuspended in LB medium to OD_600_ 0.1 and allowed to re-grow to OD_600_ 1.5. This outgrowth following the selection was done to enrich for viable cells in the population and minimize the isolation of genomic DNA from non-viable cells. Cells were harvested and genomic DNA was isolated using a cetyltrimethyl ammonium bromide protocol as described by Ausubel *et al* [2].

*MGK target DNA preparation*

DNA generated for microarray hybridization in the MGK method is referred to as ‘MGK targets’. Genomic DNA isolated from cells grown in serum (selection) and LB (reference) was used as template for preparation of MGK targets, as described by Smith *et al* [3]. The steps followed for preparation of MGK targets have been reproduced from Smith *et al*. [7].

*Linear amplification of single-stranded DNA flanking the gene-inactivation cassette or the site of transposon insertion*

Primer extension reactions were carried out in 100 μl of 1× HotMaster PCR reaction buffer (Eppendorf, Hamburg, Germany) containing 2.5 mM MgCl_2_, 0.4 mM of each deoxynucleoside triphosphate, 40 μg of genomic DNA (the equivalent of 8 ×10^9^ *E. coli* genomes), 2 pmol of each of outward-facing biotinylated primers (Up-BIO and Dn-BIO [Table S3], corresponding to upstream and downstream ends of the gene disruption cassette, respectively), and 10 U HotMasterTaq DNA polymerase (Eppendorf). The reactions were heated at 94°C for 2 minutes, followed by 15 cycles of 94°C for 30 s and 60°C for 20 s. All of the following experimental steps were performed at room temperature unless otherwise noted.

*Purification of amplified single-stranded flanks*

The amplified biotinylated flanks were separated from the genomic DNA using streptavidin-coated magnetic beads. Before use, 15 μl (150 μg) of M-270 streptavidin-coated magnetic Dynabeads^®^ (Invitrogen, Carlsbad, CA, USA) were washed twice with 200 μl binding and washing (BW) buffer (1 M NaCl, 5 mM Tris-HCl [pH 7.5], 0.5 mM EDTA). The primer extension reaction was mixed with an equal volume of 2×BW buffer, and the mixture was added directly to the washed Dynabeads and incubated for 15 minutes to allow attachment of biotinylated DNA. Beads containing biotinylated DNA flanks were separated from the supernatant using the MPC^®^-S magnetic rack (Invitrogen), re-suspended in 200 μl BW buffer, and transferred to a new microcentrifuge tube. After removal of the buffer, beads were resuspended in 200 μl of 50% formamide, and separated from supernatant using the magnetic rack. The wash with 50% formamide was repeated five times. Beads were then washed three times with 200 μl H_2_O and finally resuspended in 20 μl H_2_O (equivalent to 100 μl of primer extension reaction).

*Polyadenylation of the 3'-ends of single-stranded DNA*

The reaction was carried out in a total volume of 50 μl of buffer no. 4 (New England Biolabs, Ipswich, MA, USA; 50 mM potassium acetate, 20 mM Tris-acetate, 10 mM magnesium acetate, and 1 mM dithiothreitol) containing 0.25 mM CoCl_2_, 60 μM dATP, 20 U terminal deoxynucleotidyl transferase (New England Biolabs), and 20 μl of bead suspension from the previous step. The reaction was incubated for 1 hour at 37°C with shaking at 1000 rpm in an Eppendorf Thermomixer^®^, followed by 10 minutes heat inactivation at 75°C. Beads carrying polyadenylated DNA were separated from the supernatant, washed with 200 μl H_2_O, and resuspended in 20 μl of H_2_O.

*PCR amplification of the DNA flanks*

The polyadenylated bead-bound DNA was used as template for nested PCR amplification, with incorporation of amino-allyl dUTP allowing conjugation to fluorophores. To minimize cross-hybridization of the products amplified from the control and experimental DNA, unique mismatches were introduced into each set of nested PCR primers (Table S1). A pair of primers in each set contained one mismatch positioned five nucleotides away from the mismatch in the other set. One set of primers was used for amplification of targets to be labeled with Alexa Fluor 555, and the other set with Alexa Fluor 647 (Invitrogen). The 100 μl nested PCR reaction contained 0.2 mmol/l of each of dATP, dCTP, and dGTP; 80 μM of dTTP; 120 μM of amino allyl dUTP; 16 mM (NH_4_)_2_SO_4_; 67 mM Tris-HCl (pH 8.8); 0.01% Tween-20; 1.5 mM MgCl_2_; 5 U Taq DNA polymerase (CLP, San Diego, CA, USA); and 2 μl of bead suspension from the previous step. For the defined library, the PCR reaction mixture contained 1 μM each of the primers Up-cy3, Dn-cy3, and TATV-3 (for generating the targets to be labeled with Alexa Fluor 555). Alternatively, the mixture contained 1 μM each of the primers Up-cy5, Dn-cy5, and TATV-5 (to generate the targets to be labeled with Alexa Fluor 647). PCR conditions were as follows: 94°C for 5 minutes followed by 30 cycles of 95°C for 10 s, 47°C for 10 s, and 68°C for 10 s. Each 100 μl nested PCR reaction yielded 3 to 5 μg of target DNA product with an average size of 300 bp. Usually four to five 100 μl PCR reactions were performed. Amino-allyl modified PCR products were purified using the Wizard SV Gel and PCR Clean-up system following the manufacturer's protocol (Promega, Madison, WI, USA) and concentrated by ethanol precipitation.

*Fluorescent dye conjugation*

PCR product (10 to 20 μg) from the previous step was used for conjugation with Alexa Fluor 555 or Alexa Fluor 647 dyes, following the manufacturer's protocol (Invitrogen). Labeled products were purified with the Wizard SV Gel and PCR Clean-up system and concentrated by ethanol precipitation. DNA concentration and dye incorporation were determined using a NanoDrop^®^ ND-1000 UV/Vis spectrophotometer (NanoDrop Technologies, Wilmington, DE, USA).

*Microarray hybridization and data analysis*

Approximately 5 μg each of MGK targets prepared from each growth condition were mixed and hybridized to a custom-designed oligonucleotide microarray (Combimatrix Corporation, Mukilteo,WA) for 20 h at 50^o^C in a rotating oven [3]. Hybridization and washing steps were performed according to the manufacturer’s protocol. Microarrays were scanned using a Perkin Elmer confocal dual-laser microarray scanner equipped with ScanArray Lite software (Perkin Elmer, Boston, MA). Combimatrix Microarray Imager^TM^ software was used to obtain raw signal intensities. After background subtraction and normalization, intensity ratios (values in LB/values in serum) were calculated. Genes corresponding to probes that showed a 2-fold or greater change in intensity between the two samples were considered significant.

**Construction of gene knockout mutants in *B. anthracis***

Genomic DNA was isolated from *B. anthracis* Sterne strain using the GenElute^R^ Bacterial Genomic DNA Kit (Sigma-Aldrich) and was used as template to amplify approximately 500 bp regions upstream and downstream of each gene of interest, namely *purE*, *purK*, *carA* and *pyrC*, using primers listed in Table S3. The PCR products were cloned flanking the kanamycin resistance marker in a temperature-sensitive plasmid pKS1 [4]. Plasmids were initially transformed into *E. coli* strain GM2163 to obtain unmethylated plasmid DNA for transformation of *B. anthracis* [5]. Following introduction of the plasmids in *B. anthracis* Sterne cells, gene deletion mutants were isolated as described previously by Shatalin *et al*. [4]. Loss of pKS1-based plasmids and replacement of the chromosomal gene of interest with the kanamycin resistance marker was verified by both antibiotic susceptibility testing and PCR analysis.

**Verification of phenotypes of individual mutant strains**

Mutant *E. coli*, *S. typhimurium* or *B. anthracis* strains and the corresponding wild-type strains were grown overnight in LB medium. The next day, the cultures were diluted 50-fold in LB and grown to OD_600_ 0.6. The cells were washed with PBS and inoculated into either serum or LB medium at the following cell densities: *E. coli* and *S. typhimurium*: ~10^6^ cells/ml; *B. anthracis* : ~10^5^ cells/ml. Cultures were incubated 0, 2.5, 5, 7 and 24 h, serially diluted and plated on LB agar plates in order to determine the viable cell counts. Nucleobase-based rescue experiments were performed similarly except that serum was supplemented with either adenine or uracil to a final concentration of 1 mM. Genetic complementation experiments were also performed identically except that serum was supplemented with ampicillin for the complemented *E. coli* mutants, erythromycin for the complemented *B. anthracis* *purE* mutant and erythromycin and 1 mM IPTG for the complemented *purK*, *pyrC* and *carA* mutants of *B. anthracis*.

**Construction of plasmids for genetic complementation**

DNA fragments containing the structural regions of the *E. coli* genes *carA*, *pyrE*, *purA*, *guaB* and *purE* of with their own promoter (~ 300 bp upstream region) respectively, were PCR-amplified from the genomic DNA of the *E. coli* strain BW25113 using primers listed in Table S3. PCR products were digested with appropriate restriction enzymes and cloned into pBAD22 digested with the same restriction enzymes. Constructed plasmids were designated pCarA_EC,_ pPyrE_EC,_ pPurA_EC_, pGuaB_EC_ and pPurE_EC_ (Table S1) and used to transform the corresponding *E. coli* mutant strains. Transformants were selected on LB agar plates with ampicillin.

A shuttle vector pHT304 was used to construct complementing plasmids for the *B. anthracis* mutants [6]. The *B. anthracis* *purE* gene along with a 300 bp upstream region including its native promoter was PCR-amplified using primers listed in Table S3. The PCR product was digested with restriction enzymes *Kpn*I and *Pst*I and cloned into pHT304, digested with the same enzymes, to get the complementing plasmid pPurE_BA_. Plasmid pHTSPAC was derived from pHT304 by introducing an IPTG-inducible SPAC promoter system [7] in its multicloning site (H. Lee, unpublished). pHTSPAC was used to clone and express the *B. anthracis* genes *carA*, *pyrC* and *purK*. The structural regions of the genes were PCR amplified using primers which included a consensus *Bacillus subtilis* ribosome binding site (aaggagga) (Table S3), digested with the appropriate restriction enzymes and cloned in pHTSPAC under the control of SPAC promoter to obtain the complementing plasmids pCarA_BA_, pPyrC_BA_, and pPurK_BA_. These plasmids were isolated from the *E. coli* strain GM2163 and used to transform *B. anthracis* Sterne strain. Transformants were selected on LB agar plates supplemented with erythromycin.

**REFERENCES**

1. Baba T, Ara T, Hasegawa M, Takai Y, Okumura Y, et al. (2006) Construction of Escherichia coli K-12 in-frame, single-gene knockout mutants: the Keio collection. Mol Syst Biol 2: 2006 0008.

2. Ausubel F (1994) Current protocols in molecular biology. New York: John Wiley & Sons Inc.

3. Smith LK, Gomez MJ, Shatalin KY, Lee H, Neyfakh AA (2007) Monitoring of Gene Knockouts: genome-wide profiling of conditionally essential genes. Genome Biol 8: R87.

4. Shatalin KY, Neyfakh AA (2005) Efficient gene inactivation in Bacillus anthracis. FEMS Microbiol Lett 245: 315-319.

5. Quinn CP, Dancer BN (1990) Transformation of vegetative cells of Bacillus anthracis with plasmid DNA. J Gen Microbiol 136: 1211-1215.

6. Arantes O, Lereclus D (1991) Construction of cloning vectors for Bacillus thuringiensis. Gene 108: 115-119.

7. Yansura DG, Henner DJ (1984) Use of the Escherichia coli lac repressor and operator to control gene expression in Bacillus subtilis. Proc Natl Acad Sci U S A 81: 439-443.
