# Supplementary material for: Nucleotide Biosynthesis Is Critical for Growth of Bacteria in Human Blood
Source: PLoS Pathog. 2008 Feb 15;4(2):e37. doi: 10.1371/journal.ppat.0040037 (PMC2242838; doi:10.1371/journal.ppat.0040037)
Supplement: Supplementary file 2 [file ppat.0040037.st001.doc]

**Table S1. Plasmids and strains used in this study.**

| **Plasmid or strain** | **Relevant characteristic(s)** | **Source or reference** |
| --- | --- | --- |
| **Strains** | | |
| ***E. coli*** | | |
| BW25113 | *rrnB3 ΔlacZ4787 hsdR514 Δ(araBAD)567 Δ(rhaBAD)568 rph-1* | Baba et al*.* [1] |
| GM2163 | *F*^−^ *ara-14 leuB6 fhuA31 lacY1 tsx78 glnV44 galK2 galT22 mcrA dcm-6 hisG4 rfbD1 rpsL136 dam13::Tn9 xylA5 mtl-1 thi-1 mcrB1 hsdR2* | New England Biolabs |
| Keio library | 3985 single-gene deletions of *E. coli* K-12 strain BW25113 | Baba et al [1] |
| wt+pBAD22 | BW25113 transformed with pBAD22, Amp^R a)^ | This study |
| *carA*/pCarA_EC_ | *carA* mutant transformed with pCarA_EC_, Amp^R^ | This study |
| *pyrE*/pPyrE_EC_ | *pyrE* mutant transformed with pPyrE_EC_, Amp^R^ | This study |
| *purA*/pPurA_EC_ | *purA* mutant strain transformed with pPurA_EC_, Amp^R^ | This study |
| *purE*/pPurE_EC_ | *purE* mutant strain transformed with pPurE_EC_, Amp^R^ | This study |
| *guaB*/pGuaB_EC_ | *guaB* mutant strain transformed with pGuaB_EC_, Amp^R^ | This study |
| ***B. anthracis*** | | |
| Sterne 34F2 | pXO1^+^, pXO2^-^ derivative of Ames strain of *B. anthracis* | Hanna et al. [2] |
| wt+pHT304 | Sterne strain transformed with the pHT304 (Erm^R^) vector | This study |
| wt+pHTSPAC | Sterne strain transformed with the pHTSPAC (Erm^R^) vector | This study |
| *purE* | Sterne strain with *purE* replaced with kanamycin marker (*aphA3*) ^b)^ | This study |
| *purK* | Sterne strain with *purK* replaced with kanamycin marker (*aphA3*) | This study |
| *carA* | Sterne strain with *carA* replaced with kanamycin marker (*aphA3*) | This study |
| *pyrC* | Sterne strain with *pyrC* replaced with kanamycin marker (*aphA3*) | This study |
| *purE*/pPurE_BA_ | *purE* mutant transformed with pPurE_BA_ (Erm^R^) | This study |
| *purK*/pPurK_BA_ | *purK* mutant transformed with pPurK_BA_ (Erm^R^) | This study |
| *carA*/pCarA_BA_ | *carA* mutant transformed with pCarA_BA_ (Erm^R^) | This study |
| *pyrC*/pPyrC_BA_ | *pyrC* mutant transformed with pPyrC_BA_ (Erm^R^) | This study |
| ***S. enterica*** | | |
| SA4251 | wild-type *Salmonella enterica* serovar Typhimurium LT2 | SGSC ^b)^ |
| TT1198 | *pyrA*(-); Tet^R^, Tn10 inserted in *pyrA* | SGSC |
| TT460 | *pyrB*(-); Tet^R^, Tn10 inserted in *pyrB* | SGSC |
| TT459 | *pyrC*(-); Tet^R^, Tn10 inserted in *pyrC* | SGSC |
| TT468 | *pyrD*(-); Tet^R^ , Tn10 inserted in *pyrD* | SGSC |
| TT12910 | *pyrE*(-); Km^R^, MudJ inserted in *pyrE* | SGSC |
| TT464 | *pyrF*(-); Tet^R^, Tn10 inserted in *pyrF* | SGSC |
| TT273 | *purA*(-); Tet^R^, Tn10 inserted in *purA* | SGSC |
| TT282 | *purB*(-); Tet^R^, Tn10 inserted in *purB* | SGSC |
| TT287 | *purC*(-); Tet^R^, Tn10 inserted in *purC* | SGSC |
| TT311 | *purD*(-); Tet^R^, Tn10 inserted in *purD* | SGSC |
| TT289 | *purE*(-); Tet^R^, Tn10 inserted in *purE* | SGSC |
| TT317 | *purF*(-); Tet^R^, Tn10 inserted in *purF* | SGSC |
| TT315 | *purG*(-); Tet^R^, Tn10 inserted in *purG* | SGSC |
| TT292 | *purH*(-); Tet^R^, Tn10 inserted in *purH* | SGSC |
| TT275 | *guaB*(-); Tet^R^, Tn10 inserted in *guaB* | SGSC |
| TT278 | *guaA*(-); Tet^R^, Tn10 inserted in *guaA* | SGSC |
| **Plasmids** | | |
| pBAD22 | Amp^R^ in *E. coli* | Beckwith et al [3] |
| pKS1 | Kan^R^ Erm^R^ derived from pG-host9 and pTV1-OK, thermosensitive | Shatalin et al [4] |
| pHT304 | Amp^R^ in *E. coli*, Erm^R^ in *B. anthracis,* shuttle vector | Arantes et al [5] |
| pHTSPAC | Amp^R^ in *E. coli*, Erm^R^ in *B. anthracis*, IPTG-inducible SPAC promoter system cloned in the MCS of pHT304 | This study |
| pPurE_EC_ | *E. coli purE* + 300 bp upstream region cloned into pBAD22 between *Xba*I and *Hind*III sites | This study |
| pGuaB_EC_ | *E. coli guaB*+ 300 bp upstream region cloned into pBAD22 between *Xba*I and *Hind*III sites | This study |
| pPyrE_EC_ | *E. coli pyrE* + 300 bp upstream region cloned into pBAD22 between *Xba*I and *Hind*III sites | This study |
| pCarA_EC_ | *E. coli carA* + 300 bp upstream region cloned into pBAD22 between *Xba*I and *Hind*III sites | This study |
| pPurA_EC_ | *E. coli purA*+ 300 bp upstream region cloned into pBAD22 between *Xba*I and *Kpn*I sites | This study |
| pPurE_BA_ | *B. anthracis purE* + 300 bp upstream region cloned into pHT304 between *Pst*I and *EcoR*I sites | This study |
| pPurK_BA_ | *B. anthracis purK* cloned into pHTSPAC between *Xba*I and *Kpn*I sites | This study |
| pPyrC_BA_ | *B. anthracis* *pyrC* cloned into pHTSPAC between *Xba*I and *Kpn*I sites | This study |
| pCarA_BA_ | *B. anthracis* *carA* cloned into pHTSPAC between *Xba*I and *Kpn*I sites | This study |

Abbreviations: ^a)^ Amp^R^, ampicillin resistance; Erm^R^, erythromycin resistance; MCS, multi-cloning site; ^b)^ SGSC, Salmonella Genetics Stock Center; *aphA3*, *Enterococcus faecium* aminoglycoside phosphotransferase gene.

**REFERENCES**

1. Baba T, Ara T, Hasegawa M, Takai Y, Okumura Y, et al. (2006) Construction of Escherichia coli K-12 in-frame, single-gene knockout mutants: the Keio collection. Mol Syst Biol 2: 2006 0008.

2. Pezard C, Duflot E, Mock M (1993) Construction of Bacillus anthracis mutant strains producing a single toxin component. J Gen Microbiol 139: 2459-2463.

3. Guzman LM, Belin D, Carson MJ, Beckwith J (1995) Tight regulation, modulation, and high-level expression by vectors containing the arabinose PBAD promoter. J Bacteriol 177: 4121-4130.

4. Shatalin KY, Neyfakh AA (2005) Efficient gene inactivation in Bacillus anthracis. FEMS Microbiol Lett 245: 315-319.

5. Arantes O, Lereclus D (1991) Construction of cloning vectors for Bacillus thuringiensis. Gene 108: 115-119.
