# Supplementary material for: Nucleotide Biosynthesis Is Critical for Growth of Bacteria in Human Blood
Source: PLoS Pathog. 2008 Feb 15;4(2):e37. doi: 10.1371/journal.ppat.0040037 (PMC2242838; doi:10.1371/journal.ppat.0040037)
Supplement: Supplementary file 3 [file ppat.0040037.st002.doc]

**Table S2. Amino acid sequence similarity of the *B. anthracis* and *Homo sapiens* enzymes to the *E. coli*** nucleotide biosynthesis enzymes required for growth in blood serum.

| Purine biosynthesis | | | | |
| --- | --- | --- | --- | --- |
| Enzyme | *B. anthracis* | | *H. sapiens* | |
|  | % Identity | % Similarity | % Identity | % Similarity |
| PurA | 46 | 64 | 43 | 60 |
| PurC | 43 | 62 | 32 | 51 |
| PurD | 51 | 67 | 51 | 66 |
| PurE | 60 | 77 | 27 | 47 |
| PurF | 40 | 57 | 36 | 55 |
| PurH | 53 | 69 | 34 | 46 |
| PurK | 32 | 49 | No homologue | |
| PurL | 24 | 41 | 37 | 52 |
| PurM | 53 | 67 | 46 | 66 |
| GuaA | 52 | 69 | 37 | 52 |
| GuaB | 55 | 72 | 41 | 59 |
| Pyrimidine biosynthesis | | | | |
| Enzyme | *B. anthracis* | | *H. sapiens* | |
|  | % Identity | % Similarity | % Identity | % Similarity |
| PyrB | 37 | 54 | 43 | 62 |
| PyrC | No homologue | | No homologue | |
| PyrE | 33 | 64 | 27 | 53 |
| PyrF ^a)^ | 40 | 58 | 21 | 42 |
| CarA | 45 | 58 | 39 | 53 |
| CarB | 50 | 69 | 40 | 62 |

^a)^  The numbers shown in the table correspond to the alignment of the *E. coli* PyrF with the UMP synthase from *H. sapiens*.
