# Supplementary material for: Nucleotide Biosynthesis Is Critical for Growth of Bacteria in Human Blood
Source: PLoS Pathog. 2008 Feb 15;4(2):e37. doi: 10.1371/journal.ppat.0040037 (PMC2242838; doi:10.1371/journal.ppat.0040037)
Supplement: Supplementary file 4 [file ppat.0040037.st003.doc]

**Table S3. Primers used in this study.**

| **Primers used for MGK** | |
| --- | --- |
| **Primer name** | **Primer sequence** |
| Up-Bio | 5'-biotin-gaacttcgaactgcaggtcgac-3' |
| Dn-Bio | 5'-biotin-gtataggaacttcgaagcagctc-3' |
| Up-Cy3 | 5'-ggtcgacggacccccg-3' |
| Up-Cy5 | 5'-ggtcaacggatccccg-3' |
| Dn-Cy3 | 5'-aagcagttccagcctaca-3' |
| Dn-Cy5 | 5'-aagcagctccagtctaca-3' |
| TATV-3 or Oligo(dT9 AT15V) | 5'-T9 AT15V-3' |
| TATV-5 or Oligo(dT15 AT9V) | 5'-T15 AT9V-3' |
| **Primers used for construction of gene knockouts in *B. anthracis*** | |
| purKNotIfwdup | 5' tcta**gcggccgc**ccgttattcgttagactatgag 3' ^a)^ |
| purKPstIrevup | 5' attg**ctgcag**ttctcaattctaatgcatcatgta 3' |
| purKHindIIfwddn | 5' gcag**aagctt**atggggcatgttaatattttaaatg 3' |
| purKXhoIrevdn | 5' ttga**ctcgag**ctacgttacgtttcatttcttca 3' |
| pyrCXhoIfwdup | 5' ttga**ctcgag**atgtactctacatctgcatcca 3' |
| pyrCHindIIIrevup | 5' ttcg**aagctt**ggaaatgccaaggatggccg 3' |
| pyrCPstIfwddn | 5' gttt**ctgcag**tcctaatacatttggtaaggcg 3' |
| pyrCNotIrevdn | 5' caca**gcggccgc**tcgatcttcttaccattaaaacaagagt 3' |
| purENotIfwdup | 5' acaa**gcggccgc**ttattataataatcgttgcgataca 3' |
| purEPstIrevup | 5' ctga**ctgcag**tcccattatgactccaactagtg 3' |
| purEClaIfwddn | 5' aatg**atcgat**tacttgctgcacaaatacttgg 3' |
| purEKpnIrevdn | 5' gtaa**ggtacc**cttgattttgaaccaatctatacg 3' |
| carAKpnIfwdup | 5' tcaa**ggtacc**gaataatttcacttggtggtgtt 3' |
| carAClaIrevup | 5' ccag**atcgat**caaatgatttattcgaagatttcttaac 3' |
| carAPstIfwddn | 5' acta**ctgcag**tctccttttgccatgcgatct 3' |
| carANotIrevdn | 5' aaat**gcggccgcc**gcttcgtggaaaagaagacc 3' |
| **Primers used for construction of complementing plasmids in *E. coli*** | |
| carAcompcoliXbaIfwd | 5' tgat**tctaga**gggctttaatttttatatctgtacgtttt 3' |
| carAcompcoliHindIIIrev | 5' ccag**aagctt**gatactttttatatctgtacgtttt 3' |
| pyrEcompcoliXbaIfwd | 5' cggg**tctaga**cgtggcgctggtagatgcg 3' |
| pyrEcompcoliHindIIIrev | 5' cgct**aagctt**ggcacagttgcagtaatatgac 3' |
| purEcompcoliXbaIfwd | 5' cgca**tctaga**cgcgaatgcgcgcgaacag 3' |
| purEcompcoliHindIIIrev | 5' gcct**aagctt**tcgaatgcctgacgcagc 3' |
| guaBcompcoliXbaIfwd | 5' atct**tctaga**gaaatcgaactctaccagggc 3' |
| guaBcompcoliHindIIIrev | 5' tagt**aagctt**gggttttgttcattcatagtgc 3' |
| purAcompcoliKpnIfwd | 5' ctgc**ggtacc**ggtcaataaagccaccgcatc 3' |
| purAcompcoliXbaIrev | 5' gcaa**tctaga**acactgttcgccggc 3' |
| **Primers used for construction of complementing plasmids in *B. anthracis*** | |
| carAfwdBsRBSXbaI | 5' gtc**tctaga**aaggaggacaacaagatgaaaagacaacttatcttagaag 3' |
| carArevKpnI | 5' gatc**ggtacc**ctaggcgttttggcataactc 3' |
| pyrCfwdBsRBSXbaI | 5' gtc**tctaga**catgaattatttgtttaaaaatggtcg 3' |
| pyrCrevKpnI | 5' gatc**ggtacc**tcatactaatgcactctcctttt 3' |
| purEPstIfwd | 5' cctt**ctgcag**tatagttacaattattcttttgaat 3' |
| purEEcoRIrev | 5' ccta**gaattc**cgcctccaataatgccgattg 3' |
| purKfwdBsRBSSpeI | 5' gtc**actagt**aaggaggacaacaagatgacgagaatcatttacctggaaaaac 3' |
| purKrevKpnI | 5' gatc**ggtacc**tcatctttttccctccaacag 3' |

^a)^ The restriction enzyme sites incorporated in the sequence are shown in bold.
